# Socioeconomic, racial and ethnic differences in patient experience of clinician empathy: Results of a systematic review and meta-analysis

**DOI:** 10.1371/journal.pone.0247259

**Published:** 2021-03-03

**Authors:** Brian W. Roberts, Nitin K. Puri, Christian J. Trzeciak, Anthony J. Mazzarelli, Stephen Trzeciak

**Affiliations:** 1 Cooper University Health Care, Camden, New Jersey, United States of America; 2 Department of Emergency Medicine, Cooper Medical School of Rowan University, Camden, New Jersey, United States of America; 3 Center for Humanism, Cooper Medical School of Rowan University, Camden, New Jersey, United States of America; 4 Department of Medicine, Cooper Medical School of Rowan University, Camden, New Jersey, United States of America; University of Maryland School of Medicine, UNITED STATES

## Abstract

**Introduction:**

Empathy is essential for high quality health care. Health care disparities may reflect a systemic lack of empathy for disadvantaged people; however, few data exist on disparities in patient experience of empathy during face-to-face health care encounters with individual clinicians. We systematically analyzed the literature to test if socioeconomic status (SES) and race/ethnicity disparities exist in patient-reported experience of clinician empathy.

**Methods:**

Using a published protocol, we searched Ovid MEDLINE, PubMed, CINAHL, EMBASE, CENTRAL and PsychINFO for studies using the Consultation and Relational Empathy (CARE) Measure, which to date is the most commonly used and well-validated methodology for measuring clinician empathy from the patient perspective. We included studies containing CARE Measure data stratified by SES and/or race/ethnicity. We contacted authors to request stratified data, when necessary. We performed quantitative meta-analyses using random effects models to test for empathy differences by SES and race/ethnicity.

**Results:**

Eighteen studies (n = 9,708 patients) were included. We found that, compared to patients whose SES was not low, low SES patients experienced lower empathy from clinicians (mean difference = -0.87 [95% confidence interval -1.72 to -0.02]). Compared to white patients, empathy scores were numerically lower for patients of multiple race/ethnicity groups (Black/African American, Asian, Native American, and all non-whites combined) but none of these differences reached statistical significance.

**Conclusion:**

These data suggest an empathy gap may exist for patients with low SES. More research is needed to further test for SES and race/ethnicity disparities in clinician empathy and help promote health care equity.

**Trial registration:**

Registration (PROSPERO): CRD42019142809.

## Introduction

Empathy is sensing and detecting another’s emotions, resonating with their thoughts and feelings, and sharing and understanding their perspective. In health care, empathy is a vital clinical competency–an emotional bridge that drives compassionate care for patients [1]. As such, empathy is essential for high quality health care. Numerous studies published in the literature show that clinician empathy is associated with better patient outcomes across many different medical conditions [2–14].

Health care disparities are meaningful differences in health care quality that exist between population groups (e.g. race, ethnicity, gender, sexual orientation) not explained by variation in patient preferences, health care needs, or treatment guidelines, and often linked with socioeconomic disadvantage [15]. Although clinicians ought to have empathy for all patients, it is possible that disparities exist in empathy from clinicians. On a health care systems level, disparities in access to health care may be rooted in a societal lack of empathy for disadvantaged persons (e.g. institutionalized racism). Health care disparities occurring at the point of care with individual patients may be due to clinician bias (e.g. implicit or unconscious bias), and this may involve a lack of empathy. Examples include inadequate analgesia for Black/African American and Hispanic/Latino patients with painful conditions [16–19], inappropriately low use of cardiac catheterization for Black/African American patients with possible acute myocardial infarction [20], and clinicians’ false assumptions that Black/African American patients will have poor adherence to treatment recommendations [21], among many others. Although some studies have reported that Black/African American patients and Hispanic/Latino patients have hospital experiences that are not worse than those of white, non-Hispanic patients [22, 23], other studies have shown that race/ethnicity and SES differences exist in patient satisfaction with clinicians [24, 25], possibly due to lower quality interpersonal interactions and clinician-patient relationships [26–28], However, few data exist on SES and race/ethnicity disparities in patient experience of clinician empathy (e.g. *interpersonal* racism), specifically.

In addition, there may be important system level effects causing low SES patients to experience lower empathy from clinicians. The quality of health care services tends to vary inversely with the need for it in the population served (i.e. the “inverse care law”) [29, 30], As a result, low SES patients–who commonly have multimorbidity and the highest health care needs–often receive lower quality care, and this may include lower quality of clinician-patient relationships [31].

The Consultation and Relational Empathy (CARE) Measure is to date the most commonly used and well-validated methodology (i.e. proven reliability, internal validity and consistency [32]) for measuring clinician empathy from the patient perspective [33, 34]. The CARE Measure is a simple, rigorously tested, person-centered questionnaire that measures empathy in the context of a one-on-one therapeutic relationship between a clinician and a patient. Originally developed for assessing the empathy of primary care physicians, it has since been successfully implemented for other medical staff, allied health professionals and nurses. On a 41-point scale (range 10 [lowest] to 50 [highest]), the instrument measures a patient’s assessment of the empathy of a clinician, for example listening and understanding, being interested in the patient as a whole person, and showing compassion. The ten questions that comprise the CARE Measure are available at: http://www.caremeasure.org/CAREEng.pdf

We hypothesized that low SES patients (compared to not low SES patients) and Black/African American and Hispanic/Latino patients (compared to white, non-Hispanic patients) report lower empathy from clinicians. We aimed to test this hypothesis by conducting a systematic review and meta-analysis of all published studies containing data for patient assessment of clinician empathy using the CARE Measure.

## Methods

### Protocol and registration

We developed and published a protocol for this systematic review and meta-analysis [35]. The protocol was developed in accordance with the Cochrane Handbook [36], and reported in accordance with the Preferred Reporting Items for Systematic Reviews and Meta-Analysis Protocols (PRISMA-P) statement [37]. We report our results in this manuscript in accordance with PRISMA and the Meta-analysis of Observational Studies in Epidemiology (MOOSE) guidelines [38, 39]. We prospectively registered this systematic review in the PROSPERO international prospective register of systematic reviews (CRD42019142809). This systematic review did not collect individual patient-level data and therefore did not require ethical approval.

### Eligibility criteria

We considered any study in which patients rated their clinicians’ empathy using the CARE Measure to be eligible for potential inclusion. Our inclusion criteria were: (1) contained data for patient-reported assessment of clinician empathy using the CARE Measure; and (2) provided CARE Measure data stratified by SES and/or race/ethnicity (including attempts to contact corresponding authors to obtain stratified data, when necessary). We considered studies eligible for inclusion regardless of language if the CARE Measure was previously validated in that language. We included both observational and interventional studies. We also included abstracts if they were published in a journal. We excluded studies for which stratified data could not be obtained. We also excluded studies that did not use the original CARE Measure (e.g. used an adaptation instead), and studies in which the CARE Measure was not completed by patients (e.g. completed by surrogates). We excluded editorials, correspondence, and review papers, as well as studies that were secondary reports of previously published studies.

### Search and identification of studies

We searched the electronic databases generally considered to be the most important sources [36]: Ovid MEDLINE, PubMed, CINAHL, EMBASE, CENTRAL and PsycINFO. We also performed a supplementary search of Google Scholar. Our previously published search strategy was as follows [35]:

Ovid MEDLINE (and adapted for searching the other databases)

1. “Consultation and Relational Empathy”.mp.
2. (CARE adj3 (measure* or question* or index*)).ti,ab. and empath*.mp.
3. (CARE adj3 (measure* or question* or index*)).ti,ab. and mercer.af.
4. 1 or 2 or 3

We adopted this search strategy and search terms from a previously conducted, comprehensive and rigorous systematic review of the CARE Measure [32]. We consulted with a health librarian with expertise in systematic reviews who confirmed that the search strategy is methodologically sound. We searched from December 1, 2004 (date of the original publication of the CARE Measure) to present. We performed the search on May 21, 2020.

### Study selection and data abstraction

Two independent reviewers performed a relevance screen of the titles and abstracts of identified studies for potential eligibility. After the relevance screen, we compared the exclusion logs for the two reviewers and we calculated the Kappa statistic for assessment of interobserver agreement. In cases of disagreement, we reviewed the full manuscript for inclusion. All studies identified as potentially relevant in the relevance screen underwent full manuscript review. For each study that underwent full manuscript review, if the manuscript did not report stratified data (i.e. by SES and/or race/ethnicity) we sent an email query to the corresponding author to request stratified data. If there was no response to the initial request, we sent up to three follow-up author query emails approximately one week apart to request the data.

Using a standardized data collection form, two reviewers independently abstracted data for the following: (a) clinical context; (b) total number of patients; (c) definition of low SES (if applicable); (d) number of patients stratified by SES; (e) CARE Measure data stratified by SES (i.e. mean and standard deviation [SD]); (f) number of patients stratified by race/ethnicity; (g) CARE Measure data stratified by race/ethnicity (mean and SD). Any disagreements in the above processes were resolved by consensus with a third reviewer.

We abstracted and tabulated race/ethnicity data according to the race/ethnicity categories used in each of the included studies. To allow for pooling and comparing of data by race/ethnicity in a meta-analytic fashion we stratified abstracted data using the race/ethnicity categories for human subjects research recommended by the United States National Institutes of Health (NIH) [40]. For SES stratification, we adopted the definition of low SES used in each of the included manuscripts.

### Assessing study quality (risk of bias)

We used the Newcastle-Ottawa Scale to assess risk of bias as recommended in the Cochrane Handbook for cohort studies [41, 42]. We used the Newcastle-Ottawa Scale rather than a scale specific to clinical trials because: (a) we needed to use a single scale that could be applied to all studies in the meta-analysis; (b) as expected, the vast majority (76%) of studies in the meta-analysis were observational-only studies, not clinical trials; and (c) the Newcastle-Ottawa Scale can be applied to both observational-only studies and clinical trials. The application of the Newcastle-Ottawa Scale to clinical trials is reasonable given that what we are analyzing is exposure (e.g. SES) and outcome (CARE Measure), and allocation/randomization are not relevant for what we are studying. We deemed studies to be low risk of bias if they had seven or more stars out of a possible nine stars on the Newcastle-Ottawa Scale.

### Analysis

As recommended in the Cochrane Handbook [36], we began with a qualitative analysis. We collated studies and summarized individual study results in table format. Where possible and appropriate, we pooled data and performed a quantitative analysis with a meta-analytic approach. As described in the protocol [35], because heterogeneous populations are needed in order to assess differences between race/ethnicity or SES groups, we only performed quantitative analysis for studies that had sufficient diversity in race/ethnicity and SES in the population (defined as no single race/ethnicity or SES group comprising >90% of the study population). We used separate random effects models to calculate pooled effect sizes and report mean differences with corresponding 95% confidence intervals (CIs) for low SES versus not low SES patients, as well as all non-white versus white patients. We also used separate random effects models to make pairwise comparisons (versus white patients) for Black/African American, Hispanic/Latino, Asian, and Native American patients.

We used the I^2^ statistic to assess heterogeneity in study results for each random effects model, with the following thresholds for interpretation: low heterogeneity: 25–49%; moderate heterogeneity: 50–74%; high heterogeneity: 75% or higher [43]. We assessed for publication bias using funnel plots of the effect sizes against the precision of the studies.

Per our published protocol, we planned a sensitivity analysis restricted to studies with a low risk of bias as defined above. We also planned to analyze for possible interaction between SES and race/ethnicity, where possible, by comparing CARE Measure scores between SES categories stratified by race. We also performed post-hoc (i.e. not in our original protocol) analyses with meta-regression by year of publication, to test if there have been changes in empathy differences over time.

We used Stata 16 (StataCorp, College Station, TX) for all analyses.

## Results

**Fig 1:**
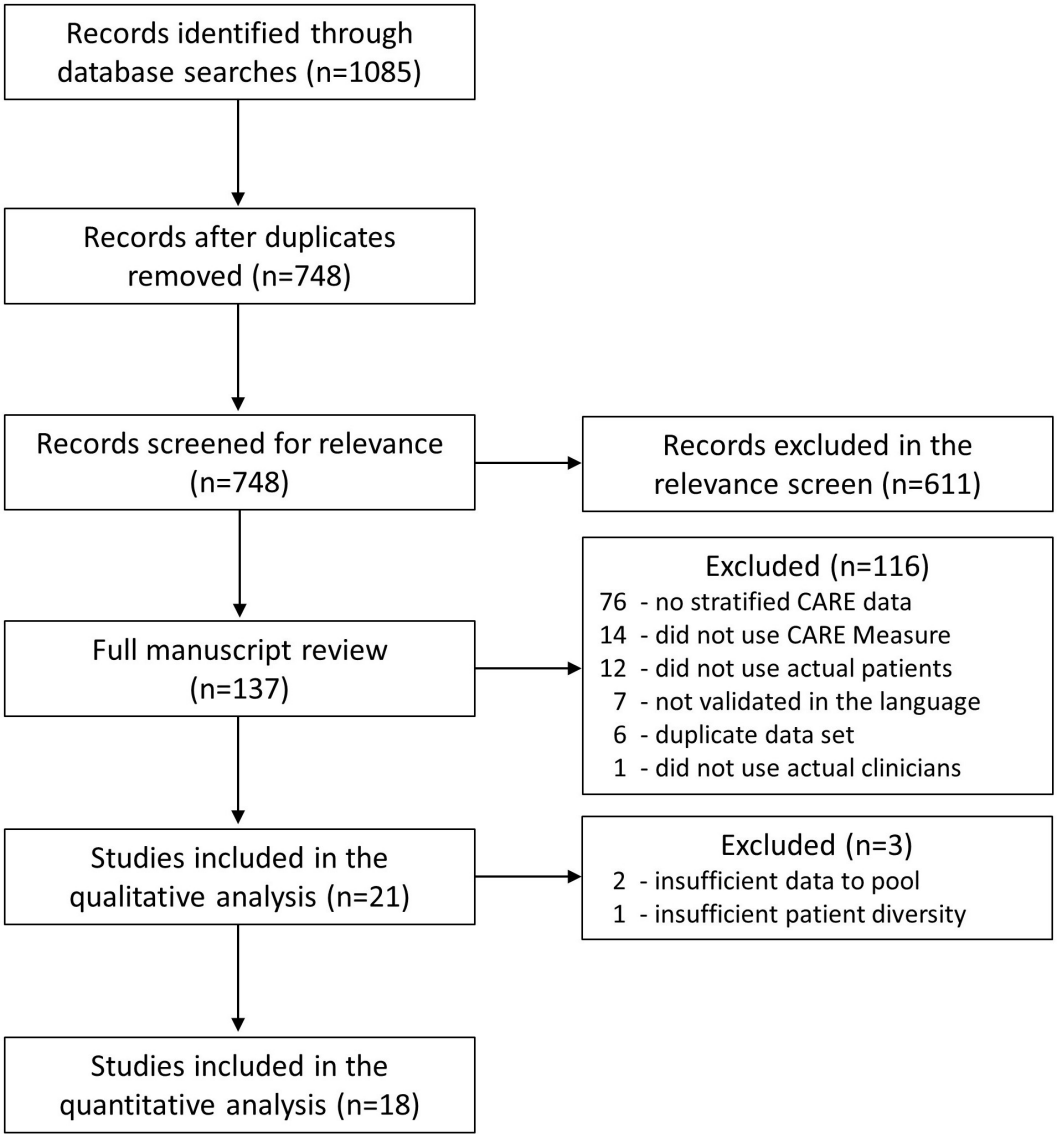
Search, inclusion and exclusion of studies flow diagram.

Our database searches yielded 1085 records. After removal of duplicates, there were 748 independent studies that underwent relevance screen. Our Kappa calculation for the relevance screen was 0.83, indicating good inter-observer agreement. Following the relevance screen, 137 studies underwent full manuscript review. Fig 1 displays the search, inclusion and exclusion of studies flow diagram.

Twenty-one studies were included in the qualitative analyses. Five studies were clinical trials and 16 studies were observational only. The countries where the studies were conducted were as follows: United States (12), United Kingdom (6), Malaysia (2), and China (1). There were no multi-national studies. The practice settings for the studies were: primary care (7), orthopedic surgery (4), multidisciplinary practice (3), sexual health (1), homeopathy (1), oral health (1), rehabilitation medicine (1), obesity medicine (1), emergency medicine (1), and oncology (1).

Three studies reported CARE Measure scores stratified by SES or race/ethnicity in the published manuscript [44–46], and the remaining 18 studies required queries to corresponding authors to obtain stratified data. Table 1 displays the nine studies with CARE Measure data stratified by SES [44–52]. Table 2 displays the 14 studies with CARE Measure data stratified by race/ethnicity [47, 51, 53–64]. Two studies had CARE Measure data stratified by both SES and race/ethnicity and thus appear in both tables [47, 51].

**Table 1:** Studies containing CARE Measure data stratified by socioeconomic status.

Author	PMID	Country	Quality^a^	Context	Low SES Definition	Results, mean CARE score (SD)
Barrett	21747102	USA	7	Primary care	Annual income <$25,000 (USD)	Low SES: n = 163, 41.9 (5.6)
						Not Low SES: n = 517, 41.5 (6.2)
Bikker	29204104	UK	5	Sexual health practice	Unemployed and seeking work	Low SES: n = 63, 47.8 (5.6)
						Not Low SES: n = 404, 48.1 (3.8)
Bikker	26493072	UK	6	Primary care	Unemployed and seeking work	Low SES: n = 39, 44.8 (6.5)
						Not Low SES: n = 165, 45.9 (6.1)
Bikker	16131282	UK	5	Homeopathy practice	Postal codes	Low SES: n = 12, 48.2 (2.9)
						Not Low SES: n = 25, 45.7 (5.5)
Hannan	31182161	USA	4	Multidisciplinary practice	Annual income </ = $49,400 (USD)	Low SES: n = 56, 36.6 (10.9)
						Not Low SES: n = 125, 41.1 (8.6)
Jani	22867682	UK	4	Primary care	Scottish Index of Multiple Deprivation (lowest versus highest quartiles)	Low SES: n = 107, 43.0 (6.8)
						Not Low SES: n = 56, 45.2 (6.8)
Mercer	15772120	UK	5	Primary care	Postal codes	Low SES: n = 1832, 40.8 (9.0)
						Not Low SES: n = 2865, 40.9 (8.6)
Mercer	26951586	UK	5	Primary care	Scottish Index of Multiple Deprivation (lowest versus highest quartiles)	Low SES: n = 356, 43.4 (6.6)
						Not Low SES: n = 302, 45.0 (6.2)
Yu	26658427	China	5	Multidisciplinary practice	Monthly income <$5,000 (HKD)	Low SES: n = 215, 33.0 (8.9)
						Not Low SES: n = 452, 34.8 (8.9)

CARE: consultation and relational empathy; SES: socioeconomic status; PMID: PubMed identification number; SD: standard deviation; USA: United States of America; UK: United Kingdom; USD: United States dollars; HKD: Hong Kong dollars.

^a^ Newcastle-Ottawa Scale.

**Table 2:** Studies containing CARE Measure data stratified by race/ethnicity.

Author	PMID	Country	Quality^a^	Context	Results, mean CARE score (SD)
Babar	28365604	Malaysia	6	Oral health practice	Chinese: n = 246, 43.3 (6.3) Malaysian: n = 12, 43.8 (4.6) Indian: n = 12, 45.0 (5.4) Other: n = 13, 46.1 (3.9)
Barrett	21747102	USA	7	Primary care	White, non-Hispanic/Latino: n = 595, 41.4 (6.0) Black or African American: n = 47, 43.0 (6.2) Hispanic or Latino: n = 18, 41.1 (6.8) Asian: n = 9, 40.9 (5.5) Native American: n = 6, 41.0 (6.3)
Hannan	31182161	USA	4	Multidisciplinary practice	White, non-Hispanic/Latino: n = 127, 40.1 (9.4) Black or African American: n = 19, 38.1 (9.4) Hispanic or Latino: n = 5, 40.4 (8.9) Other: n = 23, 39.9 (10.3)
Kootstra	29481341	USA	5	Orthopedic surgery	White, non-Hispanic/Latino: n = 108, 44.8 (7.0) Black or African American: n = 6, 46.8 (3.7) Hispanic or Latino: n = 4, 38.3 (9.2) Other: n = 6, 43.2 (6.7)
LaVela	26833180	USA	4	Rehabilitation medicine	White, non-Hispanic/Latino: n = 285, 40.3 (9.7) Black or African American: n = 76, 39.0 (10.4) Hispanic or Latino: n = 22, 42.1 (8.1) Asian: n = 3, 36.3 (17.8) Native American: n = 2, 38.5 (16.3)
Licciardone	31305871	USA	5	Primary care	White, non-Hispanic/Latino: n = 181, 38.6 (11.5) Black or African American: n = 90, 38.0 (11.0) Hispanic or Latino: n = 35, 42.0 (9.4) American Indian/Alaska Native: n = 4, 42.8 (9.2) Asian: n = 4, 38.3 (11.4) Native Hawaiian/Other Pacific Islander: n = 2, 48.5 (2.1)
McVay	30891688	USA	6	Obesity medicine	Black or African American: n = 181, 41.9 (9.7) White, non-Hispanic/Latino: n = 105, 40.7 (9.7) Hispanic or Latino: n = 44, 36.9 (9.6) More than one race: n = 8, 46.9 (4.8) Native American: n = 5, 32.0 (14.8) Other/unknown: n = 4, 46.0 (6.7) Asian: n = 2, 48.5 (0.7)
Menendez	26231482	USA	5	Orthopedic surgery	White, non-Hispanic/Latino: n = 103, 45.7 (6.9) Hispanic or Latino: n = 4, 46.0 (4.5) Black or African American: n = 2, 45.0 (7.0) Other/unknown: n = 4, 46.7 (5.8)
Moss	30911803	USA	6	Emergency medicine	White, non-Hispanic/Latino: n = 48, 42.0 (7.5) Black or African American: n = 43, 37.0 (10.8) Hispanic or Latino: n = 16, 43.0 (9.0) Other: n = 2, 39.0 (12.0)
Parker	32128909	USA	6	Oncology	White, non-Hispanic/Latino: n = 97, 45.5 (6.6) Black or African American: n = 16, 45.8 (4.5) Hispanic or Latino: n = 13, 48.0 (3.7) Asian: n = 12, 40.2 (8.3) Other: n = 2, 29.0 (14.1)
Parrish	26718069	USA	5	Orthopedic surgery	White, non-Hispanic/Latino: n = 81, 43.3 (8.3) Hispanic or Latino: n = 14, 41.8 (8.3) Black or African American: n = 4, 42.3 (7.2) Other: n = 11, 45.0 (6.5)
Weaver	31192310	USA	5	Primary care	Black or African American: n = 140, 43.4 (8.7) White, non-Hispanic/Latino: n = 102, 45.5 (6.8) Hispanic or Latino: n = 17, 43.8 (8.8) Other/unknown: n = 24, 41.3 (9.1)
Wilkens	30031600	USA	6	Orthopedic surgery	White, non-Hispanic/Latino: n = 80, 45.7 (6.4) Asian: n = 4, 45.5 (3.7) Hispanic or Latino: n = 3, 50.0 (0.0) Black or African American: n = 3, 43.3 (11.6)
Yang	31199060	Malaysia	5	Multidisciplinary practice	Malaysian: n = 65, 40.0 (7.2) Indian: n = 16, 36.3 (7.3) Chinese: n = 14, 40.3 (10.6) Other: n = 4, 34.0 (9.0)

CARE: consultation and relational empathy; PMID: PubMed identification number; SD: standard deviation; USA: United States of America.

^a^ Newcastle-Ottawa Scale.

Eighteen studies were included in the quantitative meta-analyses. Of the three studies excluded from the quantitative meta-analysis, two were excluded because there was not enough data to pool (i.e. only two studies in a Malaysian population) [53, 64] and one was excluded because there was insufficient patient diversity in the sample as defined in the methods [58]. The 18 studies in the meta-analysis included 9,708 patients in total, and 3,663 (38%) of the patients were either low SES or non-white.

**Fig 2:**
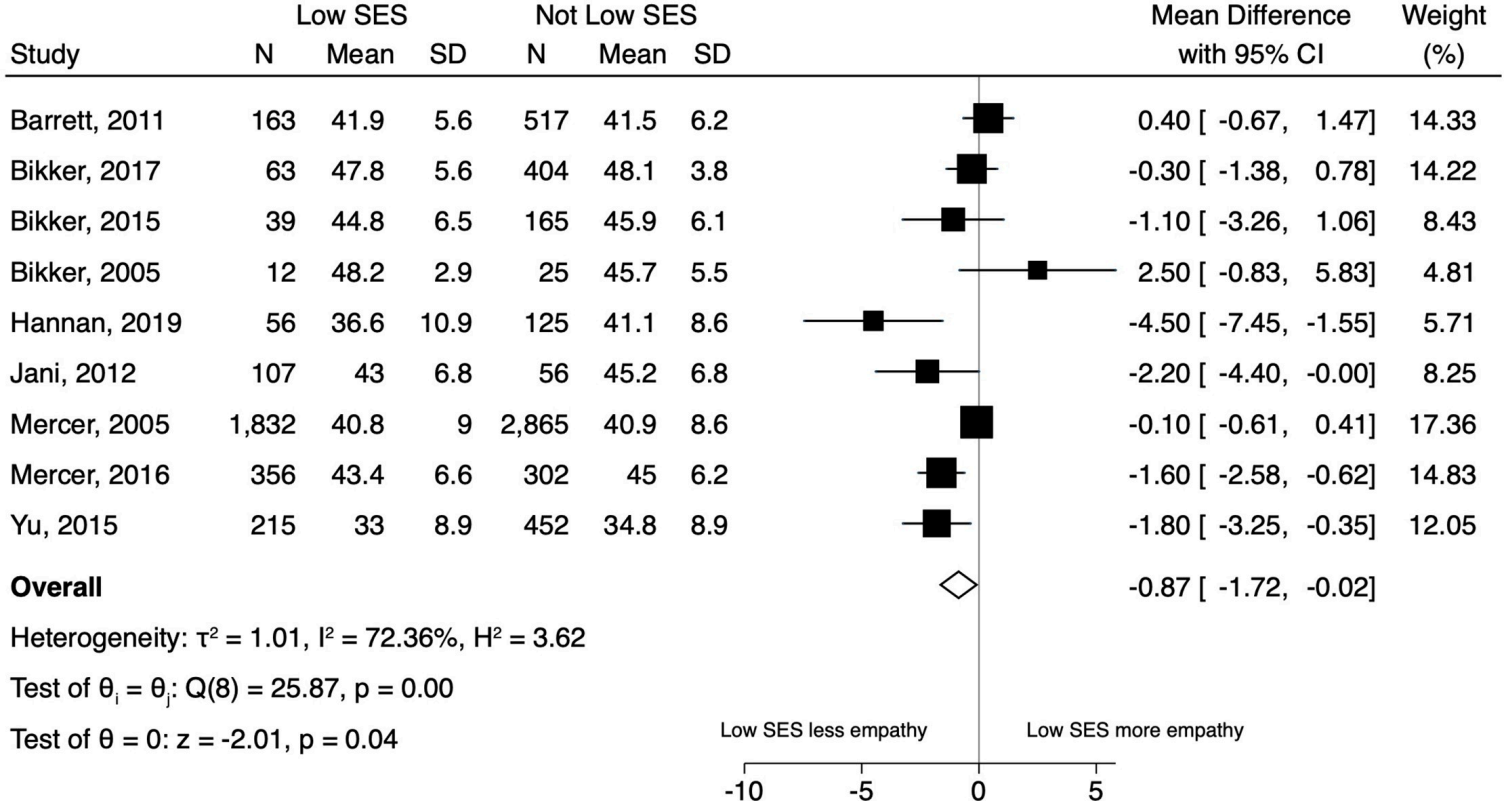
Forest plot of the results of the random effects model for patient-reported experience of clinician empathy comparing patients with low socioeconomic status (SES) to patients with not low SES.

Fig 2 displays the results of the random effects model for SES. Overall, compared to patients with not low SES, low SES was associated with lower ratings of clinician empathy (mean CARE difference = -0.87 [95% CI -1.72 to -0.02]). Heterogeneity in the model was moderate (I^2^ = 72%).

**Fig 3:**
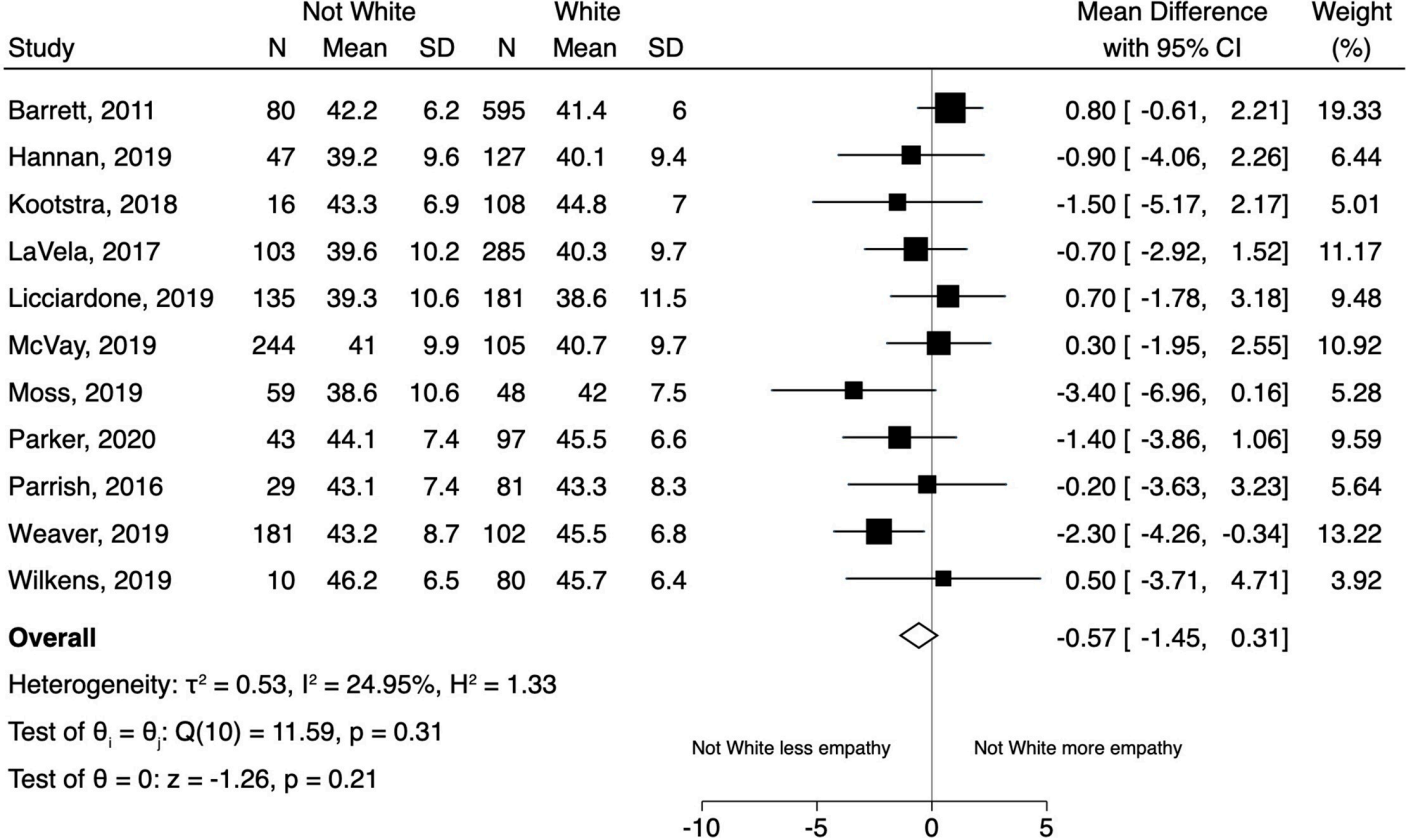
Forest plot of the results of the random effects model for patient-reported experience of clinician empathy comparing all non-white patients to white patients.

Fig 3 displays the results of the random effects model for all non-white patients compared to white patients. While we found that overall non-white patients reported lower clinician empathy compared to white patients, this difference was not statistically significant (mean CARE difference = -0.57 [95% CI -1.45 to 0.31]). Heterogeneity in the model was low (I^2^ = 25%).The results of the separate (pairwise) random effects models for Black/African American, Hispanic/Latino, Asian, and Native American patients (compared to white patients) appear in the (S1–S4 Figs). In summary, compared to white patients, empathy scores were numerically lower for patients of multiple race/ethnicity groups (Black/African American, Asian, Native American, as well as all non-whites combined) but none of these differences reached statistical significance.

In the post-hoc meta-regression by year of publication, we found that empathy differences over time were increasing for both SES and race/ethnicity (for SES: -0.18 [95% CI -0.35 to -0.02] per year comparing low SES to not low SES; for race/ethnicity: -0.22 [95% CI -0.43 to -0.01] per year comparing non-white to white patients). These data suggest that empathy differences may be widening over time.

Only one of the included studies met our definition of low risk of bias by the Newcastle-Ottawa Scale (scores for each quality domain appear in the S1 Table); therefore, we were unable to perform the sensitivity analysis restricted to low risk of bias studies. Because only two of the studies had stratified data by both SES and race/ethnicity, we also were unable to analyze for possible interaction between SES and race/ethnicity. Visual inspection of the funnel plots for the SES and race/ethnicity analyses did not suggest publication bias (S5 and S6 Figs).

## Discussion

The purpose of this systematic review and meta-analysis was to generate preliminary data for testing the hypothesis that health care disparities exist in patient experience of clinician empathy (i.e. an empathy “gap”). After quantitatively analyzing 18 independent studies of the CARE Measure including more than nine thousand patients (and nearly 40% being low SES or non-white), we found that low SES patients had significantly lower patient-reported assessments of clinician empathy compared to patients with SES that was not low. Although we did not find statistically significant differences in empathy by race/ethnicity, we point to a trend that merits further research. The CARE Measure scores were consistently numerically lower for multiple race/ethnicity patient groups (compared to white patients), including Black/African American, Asian, Native American, and all non-whites combined. In addition, none of the included studies reported significantly higher empathy for any group of non-white patients compared to whites.

We believe there is uniqueness in this report for two reasons. First, rather than testing for SES and race/ethnicity differences in patient satisfaction in the broad sense, we focused specifically on the element of clinician empathy. Second, although all health care disparities are likely rooted in a systemic lack of empathy for disadvantaged people (i.e. systems level), few data exist on disparities in individual clinician empathy during face-to-face health care encounters due to implicit bias (i.e. interpersonal level) [28].

In addition to implicit bias, there also may be important system-level factors influencing our results, especially as it relates to lower empathy for patients with low SES. According to the inverse care law, patients with the highest health care needs (such as patients with multimorbidity–who are often patients with low SES [65]) often experience lower quality care [29, 30]. Clinicians who care for low SES patients with complex needs and multimorbidity are often under-resourced, under extreme stress, and at higher risk for burnout. In addition, low SES patients may have shorter consultations with clinicians. All of these factors may contribute to patients with low SES experiencing less empathy [31].

We consider this work preliminary in nature given that there are important limitations to consider. First, the clinical significance of small differences in the CARE Measure are unclear, despite statistical significance (i.e. for SES). We are not aware of any research into the clinical significance of specific CARE Measure increments. However, our post-hoc analyses identified that such an empathy gap may be widening over time. While exploratory in nature, these post-hoc analyses provide additional scientific rationale for future studies investigating the existence of an empathy gap, as well as studies aimed at increasing clinician empathy for disadvantaged populations. Further, given recent evidence that people from low SES communities are less likely to respond to patient experience surveys resulting in non-response bias (i.e. responders are not representative of the target patient population) [66], future research needs to also focus on alternative methods to assess patient experience in disadvantaged populations so as to not underestimate possible differences in clinician empathy.

Another potential limitation is that our meta-analysis was limited to studies of the CARE Measure, and did not incorporate other previously published measures of clinician empathy. Our rationale was that we wanted to perform a quantitative meta-analysis, and this requires a single measure approach to limit heterogeneity and permit pooling of data. We selected the CARE Measure because to date it is the most commonly used assessment of clinician empathy from the patient perspective, and it has very well-validated methodology (i.e. proven reliability, internal validity and consistency) [32]. Nonetheless, it is possible that studies using a different measure would find different results [67, 68]. We also acknowledge heterogeneity (I^2^) in the random effects model for SES.

Importantly, as it pertains to the analyses by race/ethnicity, none of the studies contained information on race/ethnicity of the clinicians. Therefore, in our study it is not possible to account for race concordance/discordance (or in-group/out-group bias). We also acknowledge that the CARE Measure is a patient’s assessment of the empathy of clinicians (e.g. physicians) only. Others in the health care environment (e.g. staff, registrars, etc.) may have interactions with patients that shape patients’ experience of empathy during health care encounters in a meaningful way, and this would not necessarily be captured by the CARE Measure. We also need to acknowledge that because individual patient-level data were not collected, we could not establish our own uniform definition of low SES, and instead we relied on the definition of low SES that the authors used in each individual study. Further, only two of the studies contained data stratified by both SES and race/ethnicity so interaction could not be assessed. It is also unclear if our results reflect a true difference in clinician empathy for patients with low SES or are simply a reflection of patients who are experiencing socioeconomic deprivation. However, results from previous work refute the latter, by comparing CARE scores with objectively measured aspects of patient-centered care [45].

Lastly, we are not aware of any studies that have tested if disadvantaged persons (by either SES and/or race/ethnicity) have different expectations for clinician empathy in health care encounters due to history of discrimination (e.g. institutionalized and interpersonal racism), and this could potentially affect the results.

Finding meaningful disparities in clinician empathy would have important implications for public health because clinician empathy is vital for high quality health care. We believe the results of this systematic review and meta-analysis are important preliminary data supporting that an empathy gap may exist for disadvantaged people in face-to-face health care encounters with clinicians. More research to further test this hypothesis and help promote health care equity is warranted.

## Supporting information

S1 Checklist PRISMA 2009 checklist. (DOC)

S1 Fig Forest plot of the results of the random effects model for patient-reported experience of clinician empathy comparing Black/African American patients to white patients. (JPG)

S2 Fig Forest plot of the results of the random effects model for patient-reported experience of clinician empathy comparing Hispanic/Latino patients to white patients. (JPG)

S3 Fig Forest plot of the results of the random effects model for patient-reported experience of clinician empathy comparing Asian patients to white patients. (JPG)

S4 Fig Forest plot of the results of the random effects model for patient-reported experience of clinician empathy comparing Native American patients to white patients. (JPG)

S5 Fig Funnel plot for the random effects model of empathy and socioeconomic status (SES). (JPG)

S6 Fig Funnel plot for the random effects model of empathy and race/ethnicity (all non-whites compared to whites). (JPG)

S1 Table Assessment of the quality of each study using the Newcastle-Ottawa Scale (range 0–9 stars). (DOCX)

S1 File Data used in all analyses. (XLSX)
