# Supplementary figures and images for: Socioeconomic, racial and ethnic differences in patient experience of clinician empathy: Results of a systematic review and meta-analysis
Source: PLoS One. 2021 Mar 3;16(3):e0247259. doi: 10.1371/journal.pone.0247259 (PMC7928470; doi:10.1371/journal.pone.0247259)

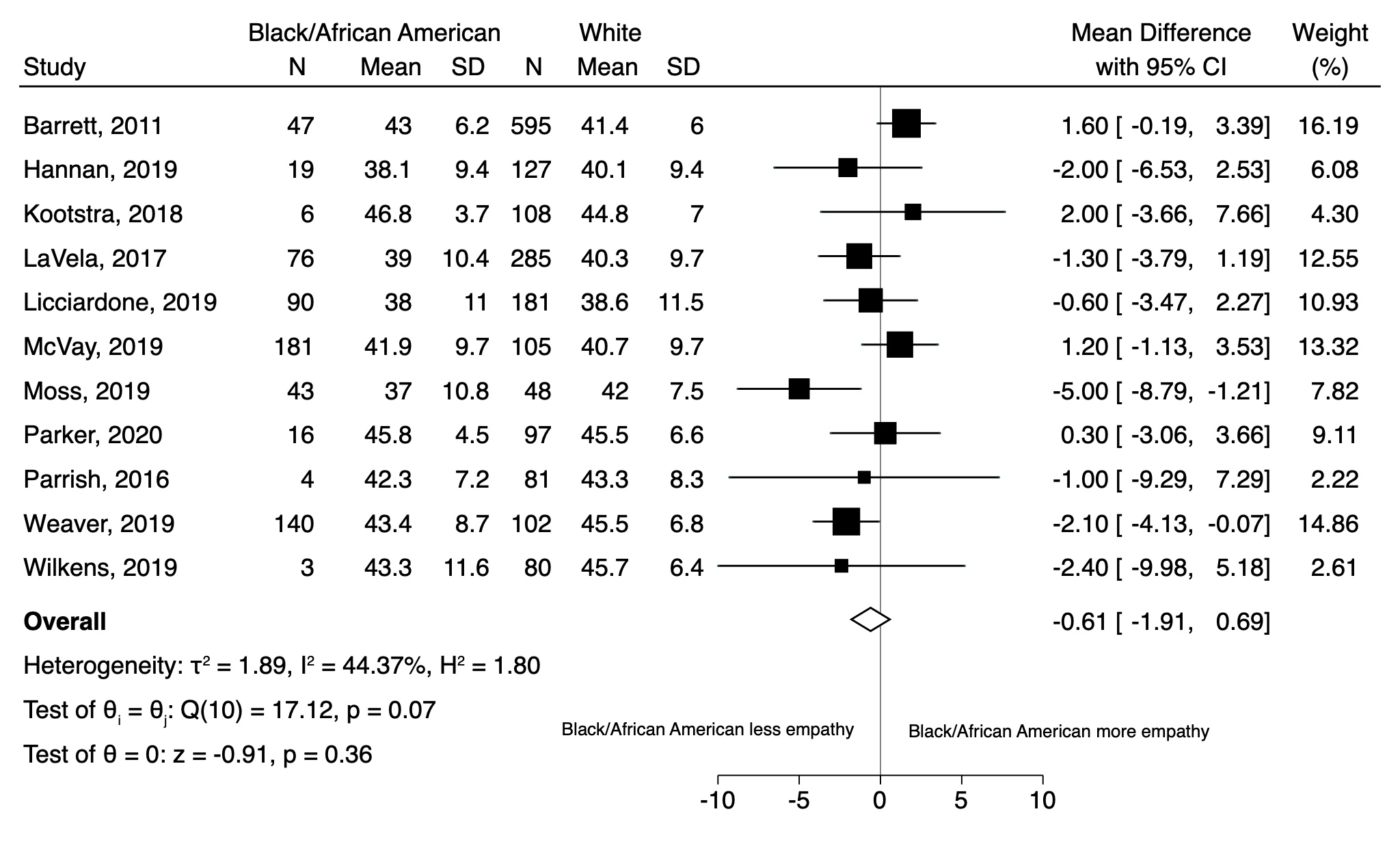

Supplement: S1 Fig — (JPG) [file pone.0247259.s002.jpg]

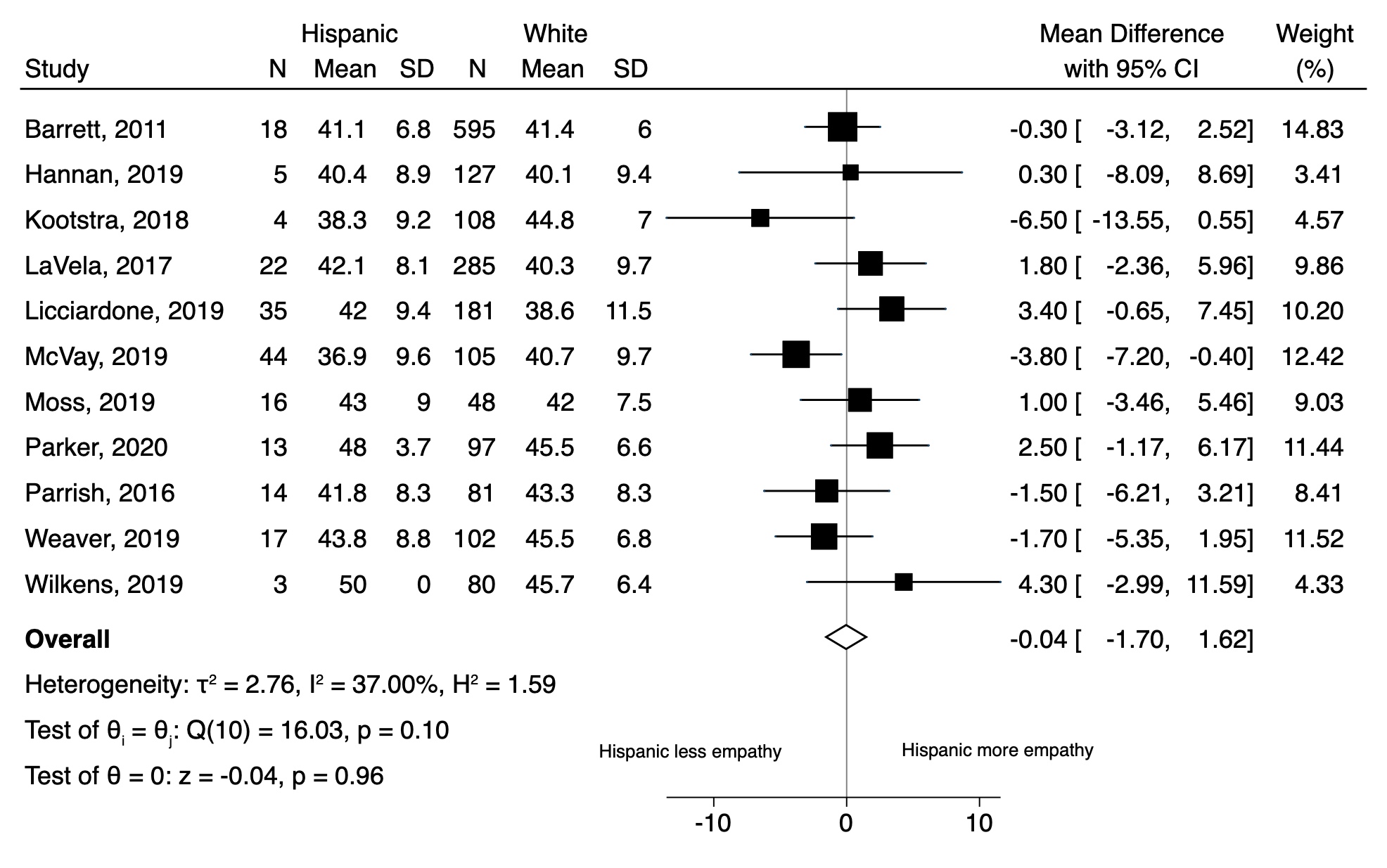

Supplement: S2 Fig — (JPG) [file pone.0247259.s003.jpg]

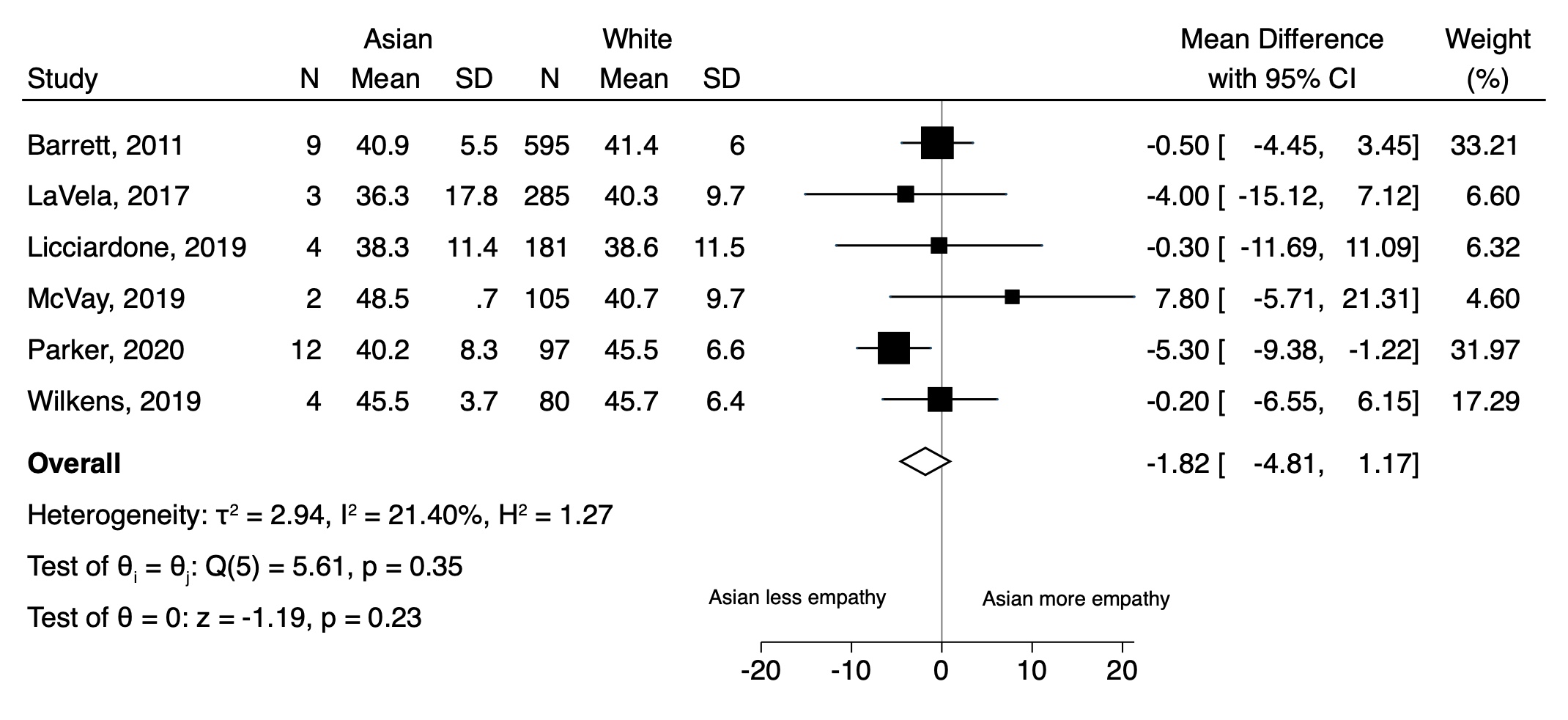

Supplement: S3 Fig — (JPG) [file pone.0247259.s004.jpg]

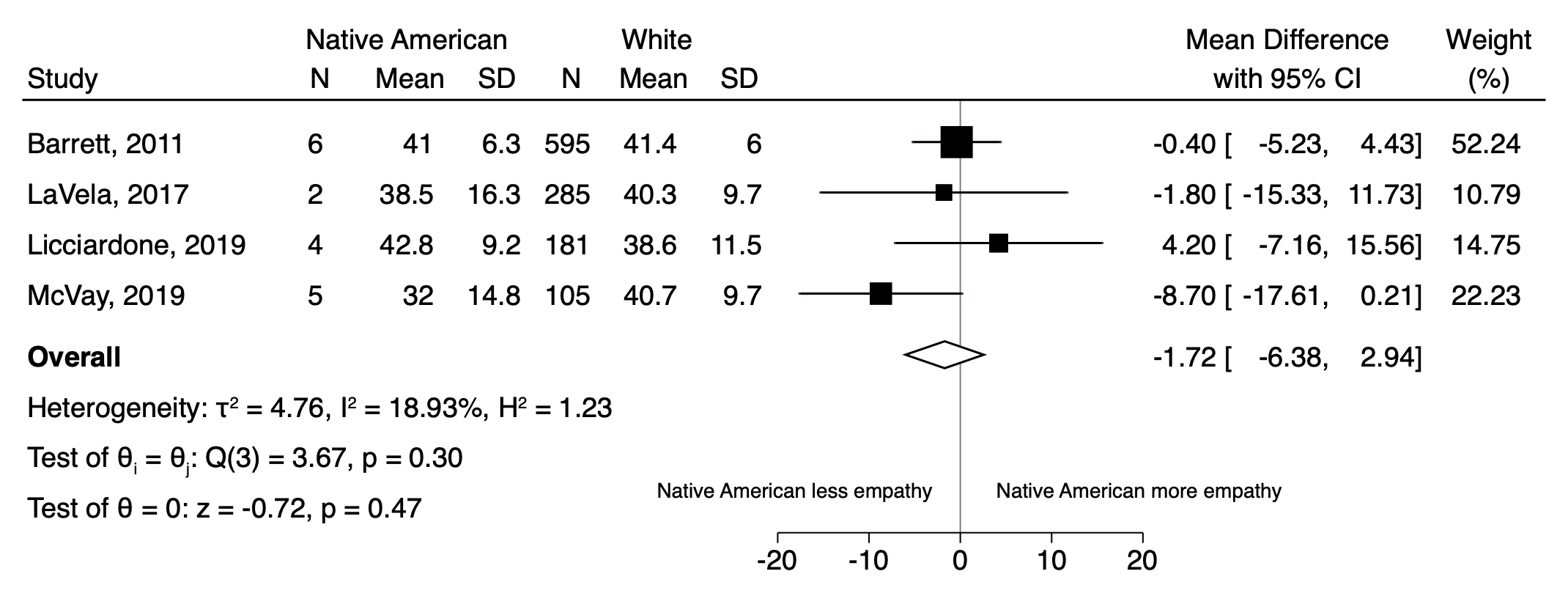

Supplement: S4 Fig — (JPG) [file pone.0247259.s005.jpg]

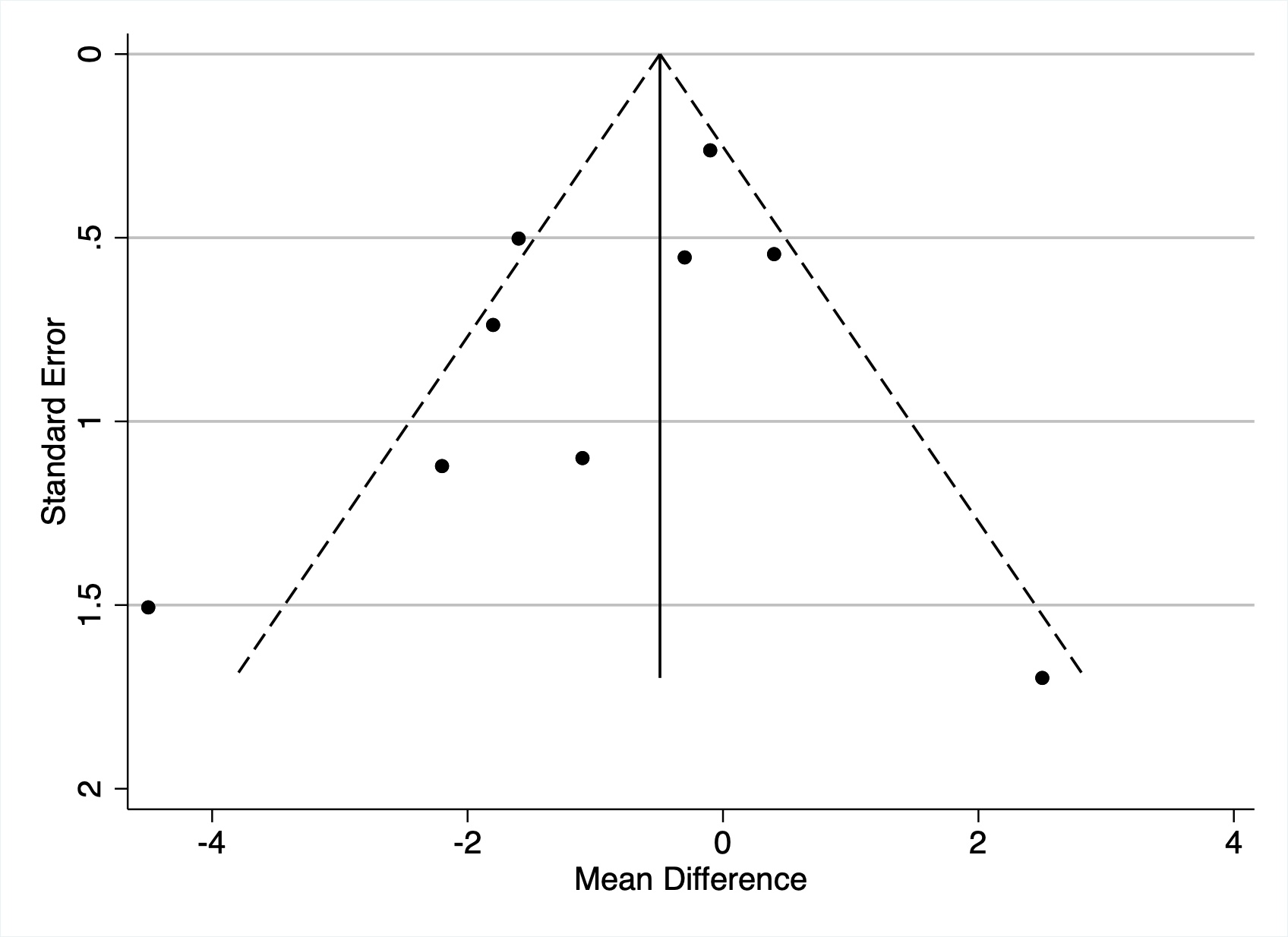

Supplement: S5 Fig — (JPG) [file pone.0247259.s006.jpg]

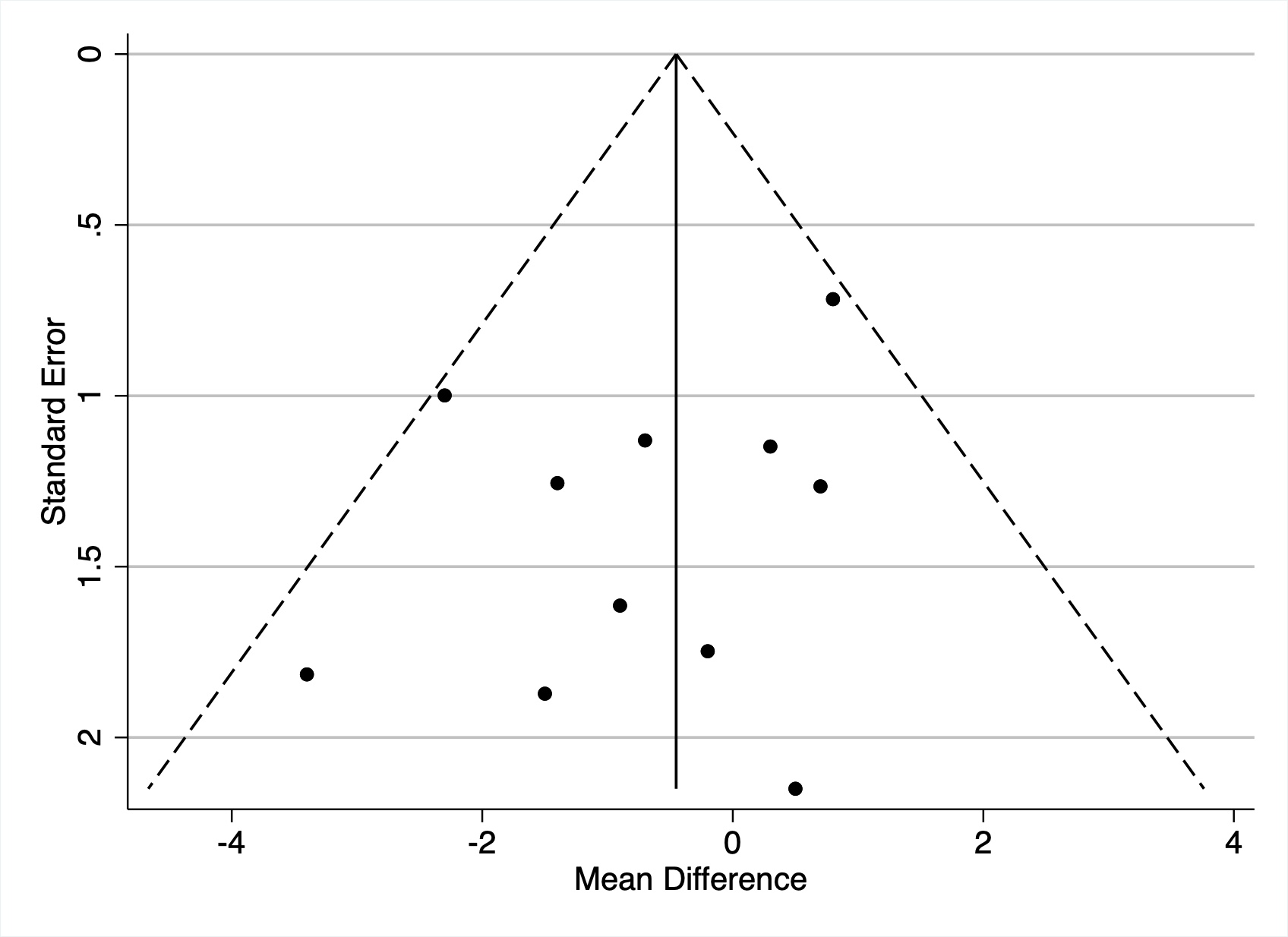

Supplement: S6 Fig — (JPG) [file pone.0247259.s007.jpg]
