# Supplementary material for: Socioeconomic, racial and ethnic differences in patient experience of clinician empathy: Results of a systematic review and meta-analysis
Source: PLoS One. 2021 Mar 3;16(3):e0247259. doi: 10.1371/journal.pone.0247259 (PMC7928470; doi:10.1371/journal.pone.0247259)
Supplement: S1 Table — (DOCX) [file pone.0247259.s008.docx]

| **Author** | **PMID** | **Selection** | **Comparability** | **Outcome** | **Total** |
| --- | --- | --- | --- | --- | --- |
| Babar | 28365604 | 3 | 1 | 2 | 6 |
| Barrett | 21747102 | 3 | 2 | 2 | 7 |
| Bikker | 16131282 | 2 | 1 | 2 | 5 |
| Bikker | 26493072 | 3 | 1 | 2 | 6 |
| Bikker | 29204104 | 2 | 1 | 2 | 5 |
| Hannan | 31182161 | 1 | 2 | 1 | 4 |
| Jani | 22867682 | 2 | 1 | 1 | 4 |
| Kootstra | 29481341 | 2 | 1 | 2 | 5 |
| Lavela | 26833180 | 2 | 1 | 1 | 4 |
| Licciardone | 31305871 | 3 | 1 | 1 | 5 |
| McVay | 30891688 | 3 | 1 | 2 | 6 |
| Menendez | 26231482 | 2 | 1 | 2 | 5 |
| Mercer | 15772120 | 2 | 1 | 2 | 5 |
| Mercer | 26951586 | 2 | 1 | 2 | 5 |
| Moss | 30911803 | 3 | 1 | 2 | 6 |
| Parker | 32128909 | 3 | 1 | 2 | 6 |
| Parrish | 26718069 | 2 | 1 | 2 | 5 |
| Weaver | 31192310 | 2 | 1 | 2 | 5 |
| Wilkens | 30031600 | 3 | 1 | 2 | 6 |
| Yang | 31199060 | 3 | 1 | 1 | 5 |
| Yu | 26658427 | 2 | 1 | 2 | 5 |
